# ADAM10 plasma levels predict worsening in cognition of older adults: a 3-year follow-up study

**DOI:** 10.1186/s13195-020-00750-y

**Published:** 2021-01-08

**Authors:** Maria Patrícia A. Oliveira Monteiro, Danielle S. M. Salheb Oliveira, Patrícia R. Manzine, Carla M. Crispim Nascimento, Ariene A. dos Santos Orlandi, Grace A. de Oliveira Gomes, Fabiana dos Santos Orlandi, Marisa S. Zazzetta, Henrique Pott-Junior, Marcia R. Cominetti

**Affiliations:** 1 Department of Gerontology, Federal University of São Carlos (UFSCar), Rodovia Washington Luís, km 235, São Carlos, SP 13565-905 Brazil; 2 Department of Nursing, Federal University of São Carlos (UFSCar), São Carlos, Brazil; 3 Department of Medicine, Federal University of São Carlos (UFSCar), São Carlos, Brazil

**Keywords:** Alzheimer’s disease, ADAM10, Biomarker, Blood, Cognition, Dementia

## Abstract

**Background:**

Blood-based biomarkers for Alzheimer’s disease (AD) are highly needed in clinic practice. So far, the gold standards for AD diagnosis are brain neuroimaging and beta-amyloid peptide, total tau, and phosphorylated tau in cerebrospinal fluid (CSF); however, they are not attractive for large-scale screening. Blood-based biomarkers allow an initial large-scale screening of patients under suspicion that could later be tested for the already established CSF biomarkers. To this regard, in this study, we evaluated whether plasma ADAM10 levels would be predictors of declines in cognition in community-dwelling older adults after a 3-year period follow-up.

**Methods:**

This was a 3-year longitudinal cohort study that included 219 community-dwelling older adults. Sociodemographic, clinical, lifestyle, depressive symptoms (GDS), and cognitive data (Mini-Mental State Examination, MMSE; Clock Drawing test, CDT) were gathered. The measurement of ADAM10 plasma levels was performed using a sandwich ELISA kit. Bivariate comparisons between groups were performed using Wilcoxon-Mann-Whitney for continuous data and Pearson’s chi-square tests with Yates continuity correction for categorical data. Longitudinal analyzes of changes in the MMSE scores were performed using linear mixed-effects modeling.

**Results:**

Baseline MMSE scores and ADAM10 levels were significantly associated with MMSE scores on the follow-up assessment. When analyzing the interaction with time, normal MMSE scores and the ADAM10 plasma levels at baseline presented a significant and independent negative association with MMSE score values on the follow-up assessment. The analyses also showed that the predictive effect of ADAM10 plasma levels on decreasing MMSE scores on follow-up seems to be more pronounced in participants with normal MMSE, when compared with those with altered MMSE scores at baseline.

**Conclusions:**

Considering that ADAM10 increase in plasma is detected as soon as in mild cognitive impairment (MCI) patients, the results presented here may support the complementary clinical use of this biomarker, in addition to the classical AD biomarkers. Taken together, these results provide the first direct evidence that changes in ADAM10 plasma levels are predictors of cognitive worsening in older adults. Moreover, this work can shed light on the study of blood biomarkers for AD and contribute to the advancement of the area.

## Background

Alzheimer’s disease (AD) is the most common type of dementia affecting older adults worldwide and is considered an important public health problem [1]. The amyloidogenic pathway of amyloid precursor protein (APP) cleavage results in the formation of β-amyloid (βA) peptide and its extracellular accumulation and aggregation in the brain is one of the causes of AD; this is known as the amyloid hypothesis of AD [2]. On the other hand, the non-amyloidogenic cleavage of APP, carried out by α and γ-secretases, avoids the βA formation.

Biomarkers for AD are highly needed in clinical practice. The amyloidogenic and non-amyloidogenic cleavages of APP are the basis for detecting of the cerebrospinal fluid (CSF) βA marker which, together with total tau (t-tau) and phosphorylated tau (p-tau), as well as neuroimaging analyses, are considered gold standards to identify the underlying pathophysiology at the earliest stages of AD. However, they do not have the scalability needed for population screening [3, 4]. Attempts to validate the aforementioned CSF biomarkers were made in blood and considerable progress was made in the field with the use of ultrasensitive high-precision platforms [5]. However, panels of markers may perform better that single candidates for diagnosing and prognosing AD [6].

Blood-based AD biomarkers are advantageous over the CSF markers due to several aspects, including, but not restricted to, their non-invasive and cost-effective screening tool characteristics [7]. However, the inter-assay variability and inconsistency of βA measurements in plasma are main factors that impair the interpretation of results and represent major obstacles to their clinical use [8]. Yet, recently promising results have demonstrated that blood p-tau181 is able to predict tau and βA pathologies and to differentiate AD from other neurodegenerative disorders [9], hence supporting this body fluid as a useful source for AD biomarker investigations, aiming to develop simple, accessible, and scalable tests for screening and diagnosis of AD.

Several blood-based AD biomarker candidates have been described, including the ADAM10 [10], which is the main α-secretase participating in the non-amyloidogenic cleavage of APP in neurons, thus having a protective function against this dementia [11]. ADAM10 can be found in different isoforms, and we have already reported that the membrane-bound platelet ADAM10 is active cleaving a fluorogenic substrate, while the isoform found soluble in plasma and CSF is inactive [12]. As a membrane-bound protein, ADAM10 acts as a sheddase cleaving different substrates on the plasma membrane, including APP in neurons, hence avoiding the production, accumulation, and aggregation of neurotoxic βA peptide [13, 14]. The levels of active ADAM10 in platelets were demonstrated to be decreased in AD patients, compared to the levels of cognitively healthy controls [15–19], whereas the levels of inactive and soluble ADAM10 in plasma were increased in MCI and AD [12]. These results are in line with most postmortem data that reveal an overall decrease of ADAM10 mRNA, protein, and/or activity in central nervous tissue of AD patients compared to age-matched controls [20].

Considering that ADAM10 is a key player and the main α-secretase involved in the non-amyloidogenic cascade, in this study, we evaluated whether its plasmatic levels would be predictors of declined cognition in community-dwelling older adults after a 3-year period follow-up.

## Methods

### Study design, participants, and setting

This was a secondary analysis of a longitudinal cohort study that used data from older adults in two time points (2015 and 2018). A convenience sample of 219 adults aged 60 years or older was recruited from a community health center in São Carlos, São Paulo, Brazil, according to the criteria detailed earlier [21]. Only complete cases were analyzed. All recruited subjects gave their written informed consent prior to their inclusion in this study. The study was conducted according to the guidelines established in the Declaration of Helsinki, and all procedures involving human subjects were approved by the Federal University of Sao Carlos’ Research Ethics Committee (number: 36167914.9.0000.5504).

### Study assessments and variables

The following sociodemographic, clinical, lifestyle, and education level data were assessed from the participants who met the inclusion criteria: sex (male, female), age (years), schooling (years), cigarette smoking (yes/no), and alcohol consumption (yes/no). Metabolic syndrome was defined considering the presence of any three of the five following metabolic impairments: elevated waist circumference, elevated triglycerides, reduced HDL-C, hypertension, and elevated fasting glucose [22].

### Depression and cognitive performance

Depression was assessed by the Geriatric Depression Scale (GDS), short version [23]. The Mini Mental State Examination [24] was used to evaluate global cognitive performance. The clock-drawing test (CDT) was applied as a more specific screening for cognitive impairment [25]. As the Brazilian population in general has a low education background, the scholarly cutoffs proposed by Brucki et al. [26] were adopted. Therefore, participants with MMSE values < 20 for illiterates, < 25 for 1–4 years of education, < 26 for 5–8 years, < 28 for 9–11 years. and < 29 for more than 11 years of formal education were considered as having altered MMSE scores. Since the mean rate cognitive impairment progression is approximately 2 to 4 points per year in the MMSE [27, 28], the 3-year follow-up period was considered enough time for detecting cognitive deterioration in this study.

### ADAM10 measurements

In the morning after an overnight fast, venous blood was drawn in tubes containing sodium citrate (3.8%) and glucose (136 mM) and centrifuged at 2400 rpm for 10 min to obtain plasma. The plasma was stored at − 80 °C until use. The measurement of ADAM10 levels in the plasma was performed using a sandwich ELISA kit (Cloud-Clone Corp., Houston, TX, USA) that contained adhered anti-human ADAM10 antibodies, which reacted with the ADAM10 present in the samples. Secondary antibodies conjugated to the alkaline phosphatase enzyme, supplied by the kit, were used to bind to the adhered proteins and, after adding substrate to the enzyme, the absorbance reading of the plates was performed on a plate reader at 450 nm wavelength (Labtec LT4000). The minimum concentration detectable by the kit is 28 pg/mL, with a detection range between 78 and 5000 pg/mL and an intra-assay coefficient of variation below 10% and inter-assay below 12%.

### Statistical analysis

Continuous data are presented as the mean (standard deviation) according to the Shapiro-Wilk test of normality. Categorical variables are presented as counts and percentages. Comparisons between groups were performed using the Wilcoxon-Mann-Whitney test for continuous variables, and Pearson’s chi-squared test with Yates’ continuity correction for categorical variables.

As the primary study outcome (MMSE score) was ascertained through two clinical assessments, patients had varying scores of MMSE captured at different times. Therefore, the longitudinal analyses of MMSE score changes over time were performed using linear mixed-effects modeling, considering the MMSE score values on follow-up as the dependent variable and incorporating the existing variability of each individual in the models (random effect).

Statistical significance was assessed at a two-sided *p* value < 0.05. All analyses were conducted using R version 3.5.3 (The R Foundation for Statistical Computing, Vienna, Austria) in R-Studio 1.1.463 (RStudio Inc., Boston, USA).

## Results

The characteristics of the participants are presented in Table 1. Only complete cases were analyzed. A total of 219 individuals were included in the study. From the 151 (68.9%) participants who had normal MMSE scores at the baseline, 23 (33.8%) progressed to altered values in the follow-up. No significant differences were found between individuals with altered and those with normal MMSE regarding the baseline and follow-up clinical or demographic variables (Table 1).

**Table 1:** Baseline and follow-up clinical and demographic parameters of the study population

Variable	Overall (***N*** = 219)	Normal MMSE (***n*** = 151)	Altered MMSE (***n*** = 68)	***p***
Age, years				0.4
Baseline	69.59 ± 7.07	69.22 ± 6.71	70.43 ± 7.81	
Follow-up	72.62 ± 7.11	72.30 ± 6.85	73.31 ± 7.67	
Female sex	127 (58.0)	86 (57.0)	41 (60.3)	0.7
Schooling				0.5
Illiterate	68 (31.1)	51 (33.8)	17 (25.0)	
1–4 years	117 (53.4)	78 (51.7)	39 (57.4)	
5–8 years	28 (12.8)	18 (11.9)	10 (14.7)	
9+ years	6 (2.7)	4 (2.6)	2 (2.9)	
Cigarette smoking, yes				0.3
Baseline	122 (55.7)	81 (53.6)	41 (60.3)	
Follow-up	119 (54.3)	79 (52.3)	40 (58.8)	
Alcohol consumption, yes				
Baseline	30 (13.7)	20 (13.2)	10 (14.7)	0.8
Follow-up	33 (15.1)	25 (16.6)	8 (11.8)	0.4
Metabolic syndrome, yes				
Baseline	96 (43.8)	65 (43.0)	31 (45.6)	0.5
Follow-up	89 (40.6)	58 (38.4)	31 (45.6)	0.1
Depression, yes				
Baseline	59 (26.9)	37 (24.5)	22 (32.4)	0.2
Follow-up	61 (27.9)	41 (27.2)	20 (29.4)	0.7
Clock-Drawing test				
Baseline				0.1
Correct	19 (8.7)	17 (11.3)	2 (2.9)	
Minimal errors	39 (17.8)	27 (17.9)	12 (17.6)	
Major errors	161 (73.5)	107 (70.9)	54 (79.4)	
Follow-up				0.1
Correct	28 (12.8)	24 (15.9)	4 (5.9)	
Minimal errors	32 (14.6)	22 (14.6)	10 (14.7)	
Major errors	159 (72.6)	105 (69.5)	54 (79.4)	
MMSE				< 0.001
Baseline	22.21 ± 4.22	24.00 ± 3.12	18.22 ± 3.56	
Follow-up	21.68 ± 4.91	22.80 ± 4.20	19.18 ± 5.44	
ADAM10, ng/mL				
Baseline	1.97 ± 1.0	2.02 ± 1.09	1.87 ± 0.93	0.4
Follow-up	2.54 ± 2.09	2.49 ± 2.06	2.64 ± 2.16	0.5

Continuous data are presented as mean ± standard deviation or median [interquartile range]. Categorical variables are presented as counts (percentage); *MMSE* Mini-Mental State Examination

To analyze the impact on ADAM10 plasma levels on the cognition of the participants, longitudinal analyses of changes in the MMSE scores over time were performed using the linear mixed-effects model considering the values of the MMSE scores in the follow-up and incorporating the existing variability in each individual in the models. Taking as a reference the model with a random effect on the intercept, it was decided to adjust different models in relation to the MMSE score values on follow-up (response variable) and the number of covariables included in the model. Table 2 shows that in the first model (model 1) that included the variables baseline ADAM10 levels (ng/mL) and baseline grouping (altered or normal MMSE) as fixed effects, having altered MMSE and higher ADAM10 levels was significantly associated with the MMSE scores in the follow-up assessment (*p* < 0.001 and 0.03, respectively). In other words, each nanogram increased in the ADAM10 plasma levels resulted in a decrease in 0.2 points in the MMSE scores of the participants in the follow-up assessment. The model 2 incorporated the variables sex (male) and age (years) besides those already included in the model 1 and the above-mentioned results remained unaltered (*p* < 0.001 and 0.02, respectively). However, in the model 3, when adjusting for baseline MMSE score values, having an altered MMSE at baseline and being male lost their significance. On the other hand, the inclusion of these variables corrected the intercept variability (Table 2).

**Table 2:** Estimates of the fixed and random parts of the models with random effect on the intercept, using MMSE score values on follow-up as the dependent variable

	Model 1			Model 2			Model 3		
**Fixed effects**	**Estimate**	**SE**	***p***	**Estimate**	**SE**	***p***	**Estimate**	**SE**	***p***
**Intercept**	24.09	0.38	< 0.001	37.73	2.33	< 0.001	10.19	1.73	< 0.001
**Age, years**	–	–	–	− 0.20	0.03	< 0.001	− 0.11	0.01	< 0.001
**Male sex**	–	–	–	0.94	0.46	0.04	0.18	0.25	0.4
**Baseline altered MMSE**	− 5.04	0.54	< 0.001	− 4.79	0.49	< 0.001	0.60	0.35	0.08
**ADAM10, ng/mL**	− 0.2	0.1	0.03	− 0.2	0.1	0.02	− 0.2	0.07	0.003
**Baseline MMSE score**	–	–	–	–	–	–	0.90	0.03	< 0.001
**Random effects**	**Variance**	**SD**		**Variance**	**SD**		**Variance**	**SD**	
**Individuals (intercept)**	9.38	3.06		7.21	2.69		0.0	0.0	
**Residuals**	7.01	2.65		7.01	2.65		6.02	2.45	
**Bayesian information criterion**	2175			2148			1871		

*SE* standard error, *SD* standard deviation, *MMSE* Mini-Mental State Examination

**Fig. 1:**
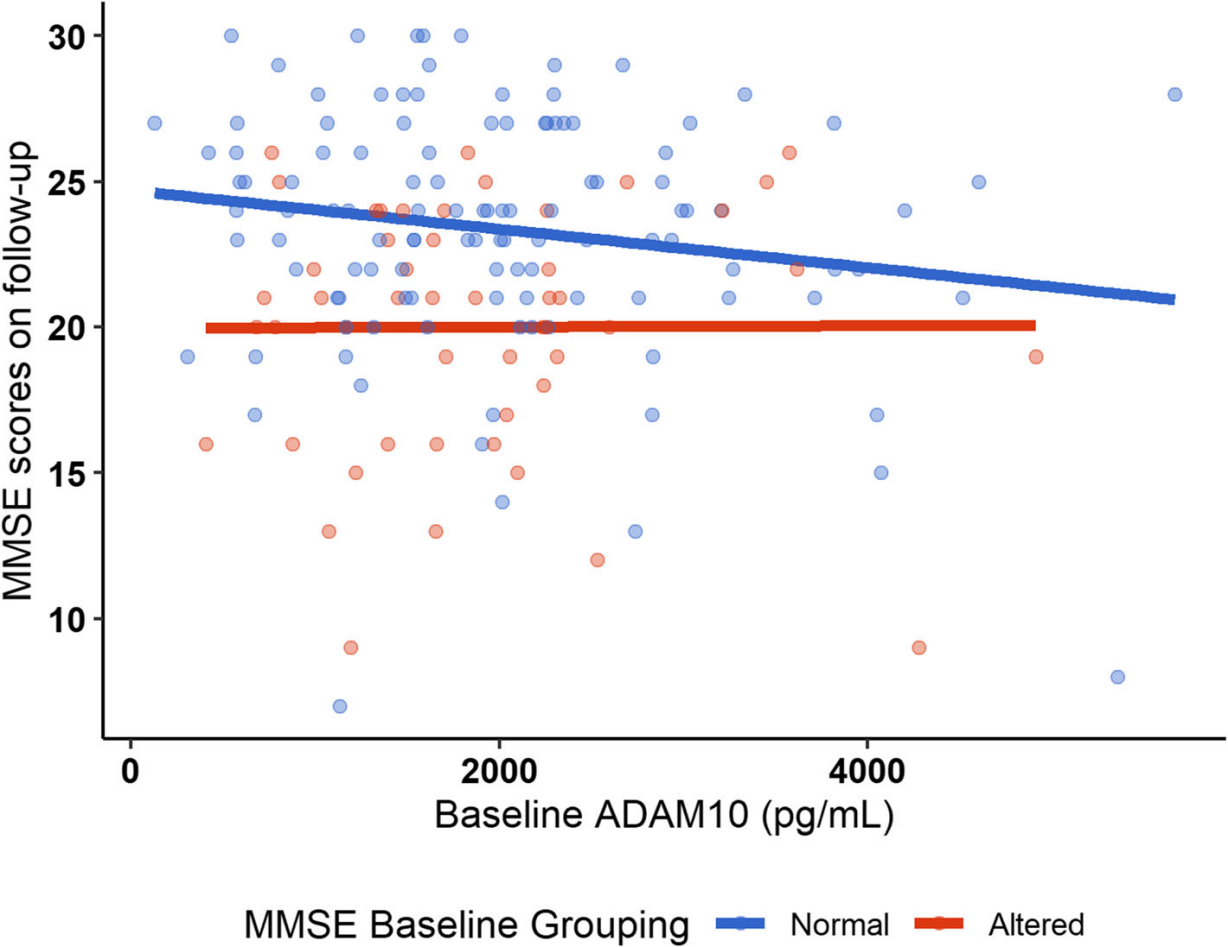
Distribution of the participants according to the baseline ADAM10 plasma levels and MMSE scores on follow-up. Red circles: participants with altered MMSE at baseline; blue circles: participants with normal MMSE at baseline. The bold blue line indicates the regression line estimated for those with normal MMSE at baseline, whereas the bold red line indicates the regression line estimated for those with altered MMSE at baseline

In model 4, we investigated further if there was an interaction of the baseline grouping (altered or normal MMSE) with ADAM10 plasma levels, adjusting for the time of assessment (Table 3). Therefore, model 4 included the interaction of the altered or normal MMSE scores, ADAM10 levels, and baseline MMSE scores. Model 5 included, besides the aforementioned variables, also the variables age (years) and sex (male). Results show that the interaction term between the baseline grouping (altered or normal MMSE) and ADAM10 plasma levels was statistically significant (*p* = 0.001), that is, the increase of each nanogram of ADAM10 in plasma remained to produce a decline of 0.2 points in the follow-up MMSE scores in those with normal MMSE at baseline (Table 3). Therefore, for this specific population, in participants with normal MMSE scores at baseline evaluation, higher ADAM10 plasma levels predict worsening in cognition, as demonstrated by lower scores in MMSE in the follow-up assessment (Fig. 1).

**Table 3:** Estimates of the fixed and random parts of the models with interaction of the baseline grouping with ADAM10, and random effect on the intercept, using MMSE score values on follow-up as the dependent variable

	Model 4			Model 5		
**Fixed effects**	**Estimate**	**SE**	***p***	**Estimate**	**SE**	***p***
**Intercept**	1.72	0.82	0.03	10.93	1.60	< 0.001
**Age, years**	–	–	–	− 0.11	0.01	< 0.001
**Male sex**	–	–	–	0.17	0.25	0.4
**Altered MMSE: ADAM10 (ng/mL)**	− 0.08	0.1	0.4	− 0.1	0.1	0.2
**Normal MMSE: ADAM10 (ng/mL)**	− 0.2	0.08	0.001	− 0.2	0.08	0.001
**Baseline MMSE score**	0.93	0.03	< 0.001	0.88	0.03	< 0.001
**Random effects**	**Variance**	**SD**		**Variance**	**SD**	
**Individuals (intercept)**	0.0	0.0		0.0	0.0	
**Residuals**	6.64	2.58		6.0	2.45	
**Bayesian information criterion**	1944			1921		

*SE* standard error, *SD* standard deviation, *MMSE* Mini-Mental State Examination

Finally, the relation of MMSE scores with ADAM10 levels was further analyzed using two multivariate linear regression models (Table 4). In baseline, MMSE scores were independently associated with age (years) and an altered MMSE baseline classification, whereas on follow-up, male sex also became significantly associated, besides age and an altered MMSE in the follow-up classification. Moreover, the follow-up model also revealed a trend of increasing ADAM10 levels and decreasing MMSE score (*p* = 0.06).

**Table 4:** Results for the multivariate linear regression model using baseline and follow-up MMSE scores as the dependent variable

**Baseline**			
**Dependent variable**	***R***^**2**^	**Adjusted** ***R***^**2**^	***p***
MMSE baseline score	0.47	0.46	< 0.001
Baseline independent variables	Unstandardized β coefficient	SE	
Intercept	30.79	2.27	< 0.001
Age, years	−0.09	0.03	0.003
Male sex	0.81	0.45	0.07
Altered MMSE	−6.02	0.48	< 0.001
ADAM10, ng/mL	−0.1	0.2	0.4
**Follow-up**			
**Dependent variable**	***R***^**2**^	**Adjusted** ***R***^**2**^	***p***
MMSE follow-up score	0.54	0.53	< 0.001
Follow-up independent variables	Unstandardized β coefficient	SE	
Intercept	38.97	2.63	< 0.001
Age, years	− 0.20	0.03	< 0.001
Male sex	1.52	0.49	0.002
Altered MMSE	− 6.12	0.52	< 0.001
ADAM10, ng/mL	− 0.2	0.1	0.06

*MMSE* Mini-Mental State Examination, *SE* standard error

## Discussion

This work shows, using the linear mixed-effects modeling, that increases in ADAM10 plasma levels lead to a deterioration in cognition, as demonstrated by the decreasing in MMSE scores after a 3-year period follow-up, evidencing that ADAM10 plasma levels can predict declines in cognitively healthy older adults.

In previous studies, we and others have shown that levels of membrane-bound ADAM10 are reduced in platelets of patients with AD compared to cognitively healthy individuals [16, 17, 19] and that this reduction correlated with patients’ cognitive performance, as measured by the CDT [29] or MMSE [15] scores. Moreover, levels and platelet ADAM10 activity were shown to be increased throughout cognitively healthy aging, pointing to the possibility that ADAM10 might contribute to or is a prerequisite for cognitively healthy aging [30]. On the other hand, ADAM plasma levels were found to be increased as early as in patients with mild cognitive impairment (MCI), as well as in AD, compared to healthy controls [12]. We hypothesized that these higher plasmatic ADAM10 levels found in MCI and AD patients represent less active protein bound at the platelet’s membrane exerting the sheddase activity. This could also be the case of neuronal ADAM10, where inactive forms can be cleaved from the membrane and released in the CSF by other proteins.

In agreement with this hypothesis, ADAM10 itself can undergo shedding and be extracellularly released by other proteins from the ADAM family, ADAM9 and 15 [31], which can be the source of the plasmatic detection of this protein. In addition, recent findings of our group have demonstrated that in plasma and CSF samples of both healthy and AD patients, ADAM10 is unable to cleave a fluorogenic substrate, whereas in whole lysates of platelets and SH-SY5Y neuroblastoma cells, the protein is active [12].

The requirement of a membrane-bound form for ADAM10 activity was further highly supported by findings of a study showing that only the active form of this metalloproteinase is expressed at the surface of different cell types, including leukocytes derived from peripheral blood [32]. Moreover, the negatively charged phospholipid phosphatidylserine (PS) translocation to the outer membrane leaflet is pivotal for ADAM10 to exert its sheddase function [33]. Nevertheless, a mechanism of alternative splicing that produces a different ADAM10 isoform or a regulation mechanism performed by endogenous inhibitors, such as tissue inhibitors of metalloproteinases (TIMPs) [34], cannot be ruled out. Mass spectrometry analyses of the ADAM10 sequence would help to identify this isoform and should be a matter for further studies.

In previous studies, we demonstrated that the levels of ADAM10 in platelets had sensitivity and specificity of 80 and 91% respectively, to identify AD patients versus controls matched by sex and age [15]. These experiments were performed in platelets, where we have shown that the protein is active, as it is bound to the membrane. When considering plasmatic ADAM10, the protein achieved 72% sensitivity and 100% specificity, at the cutoff > 1.8 ng/mL, to correctly differentiate among healthy controls versus MCI and AD patients [12].

Here, we used different models to investigate whether the plasmatic levels of ADAM10 would be efficient to predict cognitive declines in older adults after a 3-year follow-up period. We showed that the increase in ADAM10 plasma levels influences the decrease of the MMSE score values in the follow-up, and this seems to be more significant in those with normal MMSE at baseline, therefore proving that ADAM10 plasma levels can be a predictor of cognitive decline. On the other hand, in patients with already altered MMSE scores at baseline, ADAM10 did not act as a predictor of worse cognition in the follow-up assessment. Thus, the measurement of ADAM10 in patients with suspected cognitive decline, but who have not yet reached such a decline, may allow early interventions that could retard or even prevent the AD onset.

## Limitations

It is important to highlight that MMSE is a screening tool for cognitive impairment that detects losses in the evolutionary follow-up of dementias [24]. However, in some populations, individuals with lower educational levels perform worse than individuals from countries with high levels of education, but still have no cognitive decline. Regarding this, MMSE cutoffs were validated for each population, including the Brazilian one [26, 35]. Hence, the results found here may not represent the general population and should be adapted for different specificities, such as the education level.

Other limitations of this work include the evaluation of a single AD blood biomarker candidate, instead of a panel or a signature that would be more representative of the longitudinal changes in cognition. Moreover, a lack of a complete battery including the application of a diverse set of instruments does not allow a detailed cognitive evaluation of the participants. In addition, and as the study was not designed for this purpose, we were not able to specify a cutoff point value for ADAM10 levels to differentiate participants that would have future decline in cognition from those who will remain cognitively healthy. For this, additional studies with longer follow-up periods and higher sample sizes will be necessary. Yet, this is the first longitudinal study investigating the effects of plasmatic ADAM10 level changes on cognition.

## Conclusions

The results presented here provide the first direct evidence that changes in ADAM10 plasma levels can predict cognitive worsening in older adults, supporting its complementary clinical use for the AD diagnosis, in addition to the classical CSF-based biomarkers. This work can shed light on the study of blood-based AD biomarkers, open up new possibilities for investigations, and contribute to the advancement of the field.

## Data Availability

The datasets used and analyzed during the current study are available from the corresponding author on reasonable request.

## Abbreviations

α2M: Alpha-2-macroglobulin
βA: β-Amyloid peptide
AD: Alzheimer’s disease
ADAM10: A disintegrin and metalloproteinase
ApoA-1: Apolipoprotein A-1
APP: Amyloid precursor protein
BBB: Blood-brain barrier
CDT: Clock Drawing test
CSF: Cerebrospinal fluid
ELISA: Enzyme-linked immunoassay
GDS: Geriatric depression scale
IGFBP-2: Insulin growth factor binding protein-2
MCI: Mild cognitive impairment
MMSE: Mini-Mental State Examination
p-tau: Phosphorylated tau
PP: Pancreatic polypeptide
PS: Phosphatidylserine
t-tau: Total tau
